# Interfacial Reaction Mechanism between Ceramic Mould and Single Crystal Superalloy for Manufacturing Turbine Blade

**DOI:** 10.3390/ma15165514

**Published:** 2022-08-11

**Authors:** Jiansheng Yao, Longpei Dong, Zhenqiang Wu, Lili Wang, Bin Shen, Xiaowei Yang

**Affiliations:** Science and Technology on Advanced High Temperature Structural Materials Laboratory, AECC Beijing Institute of Aeronautical Materials, Beijing 100095, China

**Keywords:** DD6 single crystal superalloy, Y_2_O_3_ ceramic mould, interfacial reaction

## Abstract

Single crystal superalloys are the preferred materials for manufacturing turbine blades of advanced aero-engines, due to their excellent high temperature comprehensive performance. The interfacial reaction between alloys and ceramic mould are an important factor to influence the surface quality and service performance of the turbine blade. It is very important to reveal the interfacial reaction mechanism to improve turbine blade quality and yield rate. In this paper, the interfacial reactions between DD6 single crystal superalloy and ceramic mould were investigated by scanning electron microscopy (SEM), energy dispersive spectroscopy (EDS) and X-ray diffraction analysis (XRD). The results show that the main reaction products were HfO_2_, Al_2_O_3_ and Y_3_Al_5_O_12_ when the yttrium oxide powders were the prime coat materials, while alloy surface suffered undesirable sand fusion; the thicknesses of the reaction layers were over 20 μm. The reaction layer can be divided into two layers, the layer close to the alloy was mainly composed of Al_2_O_3_ and Y_3_Al_5_O_12_, and the layer close to the mould was composed of SiO_2_, Al_2_O_3_ and Y_3_Al_5_O_12_. Avoiding the formation of Y_2_O_3_-Al_2_O_3_-SiO_2_ ternary low-melts can solve the interfacial reaction between DD6 alloy and yttrium oxide mould.

## 1. Introduction

Nickel-based superalloys have suitable temperature bearing capacity and high-temperature all-around performance and are the preferred materials for the preparation of turbine blades of aircraft engines [1,2,3,4], and precision casting technology is an essential means of its manufacturing. Ceramic mould preparation technology is one of the critical technologies in the precision casting process, and it is the key to ensure the shape of the blade and achieve near-net type and no-margin casting. With the increasing complexity of the design of superalloy components and the more demanding process requirements, the interfacial reaction between superalloy melts and ceramic moulds has become one of the critical factors affecting the yield of turbine blade development and has gradually become a research hotspot for researchers [5,6,7,8,9,10,11,12]. As we all know, the degree of interfacial reaction between the ceramic mould and the alloy is highly dependent on the chemical stability of the primary material. In order to inhibit the interfacial reaction, it is crucial to choose suitable primary material that has excellent chemical stability. Common primary materials are mainly white fused alumina [13], mullite (EC95) [14], zircon powder [15], cobalt aluminate [16], zirconia [17], yttrium oxide [18], etc., of which white fused alumina and mullite are generally used for directional solidification, single-crystal castings; zircon powder and cobalt aluminate are generally used for equiaxed crystal castings; and zirconia and yttrium oxide are generally used for highly active titanium alloys. Due to the higher refractory resistance and chemical stability of yttrium oxide, it is possible to solve the problem of the interfacial reaction between single crystal superalloy and mould material without considering cost factors.

Some researchers have reported several interfacial reactions between nickel-based superalloy and different ceramic moulds, and results indicated that the elements of Al [19], Cr [20], C [21] and Hf [8] in the superalloy exhibited the highest reactivity, which caused the major interfacial reactions. Li Qing et al. [22] concluded that the contents of active elements and the pouring temperature were the main factor which would accelerate the chemical reaction between nickel-based superalloys and ceramic mould; the reaction products were HfO_2_ and Al_2_O_3_ if the alloys containing hafnium, but for alloys without hafnium, the reaction product was mainly Al_2_O_3_. For example, only Al_2_O_3_ was found on the interface between ceramic moulds and DD6 alloy, which is with low hafnium content, when the pouring temperature was up to 1570 °C [23]. However, there was no research on the interface reaction between Y_2_O_3_ mould and DD6 single crystal superalloy.

DD6 single crystal superalloy is the second generation of single crystal superalloy independently developed in China [24,25,26], which has the advantages of high-temperature strength, good comprehensive performance, stable structure and good casting process performance [27,28]. This alloy can fulfil the condition of high-quality complex shapes and good surface finish, as well as thin walls in order to achieve good thermal dissipation and other advantages. During the casting process of DD6 single crystal alloy, the interfacial reaction between the ceramic mould and the alloy is a fundamental reason for surface sand burning defects and internal inclusions of the turbine blades. In this work, the yttrium oxide powder was selected as the primary materials, the white fused alumina was used as the backup layer materials, the mould was prepared according to the coating process. The second-generation single crystal alloy DD6 was used for casting and sampling. The composition, morphology and thickness of the reaction layer of the interface yttrium oxide prime layer and the DD6 alloy were analysed, the interfacial reaction mechanism was studied, and the possibility of yttrium oxide as a prime material of ceramic mould was discussed, in order to solve the interfacial reaction problem in the casting process. The research provides an essential theoretical basis and technical support for improving the surface quality of turbine blades.

## 2. Materials and Methods

In this experiment, DD6 single crystal superalloy and yttrium oxide powder were used as the prime material for the casting test of the ceramic mould. The chemical composition of DD6 single crystal alloy is shown in Table 1 [29].

In the experiment, the ceramic mould was made by the standard procedure in the investment casting process. Both the prime and backup slurries used to make the ceramic mould were composed of refractory material and liquid binder. The 320 mesh (D50 is about 20 μm) yttrium oxide powder was used as the prime materials, and the 200 mesh (D50 is about 30 μm) white fused alumina powder was used as the backup materials. The liquid binder was colloidal silica which contained 30 wt.% SiO_2_. Some polymers or fibres can be added into the colloidal silica as a reinforcing phase [30], and in this study, we only use the colloidal silica with polymers for the backup slurry. The main components of yttrium oxide powder and white fused alumina powder are shown in Table 2 and Table 3. The physical and chemical properties of colloidal silica are shown in Table 4. A wax pattern was dipped into the prime slurry which made by yttrium oxide powder and colloidal silica and sprinkled with refractory stucco and dried. After drying, the pattern was dipped into the backup slurry which made by white fused alumina powder and colloidal silica and sprinkled with refractory stucco and dried. The backup dipping–drying process repeated 5 times to form a 6-layer ceramic mould. After de-waxing by high-pressure steam and sintering at 950 °C, DD6 alloy was cast and cooled in a vacuum directional solidification furnace according to the casting process. The casting temperature was 1530 °C, and the pulling rate was 3.0 mm/min. After the plate was cooled to room temperature, the interfacial reaction analysis samples were extracted.

**Figure 1:**
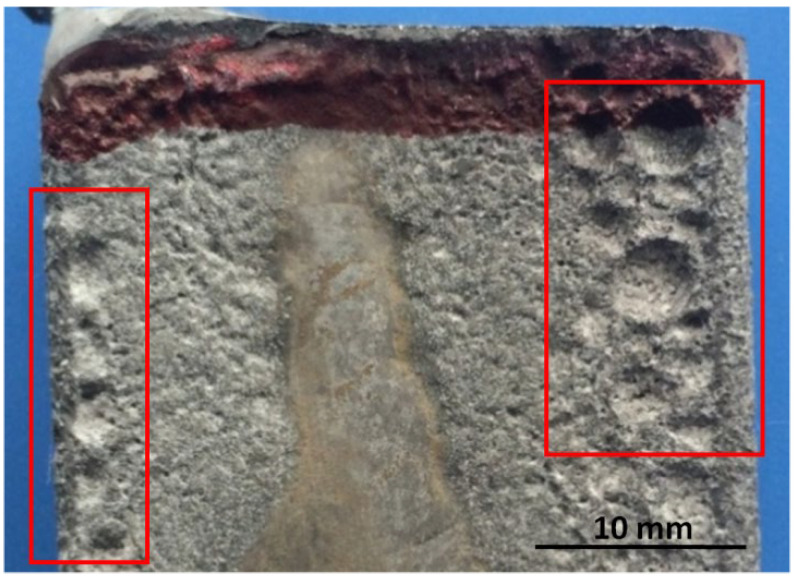
The surface morphology of DD6 casting by Y_2_O_3_ mould.

The morphology of the interfacial reaction layer between superalloy and the ceramic mould was analysed by scanning electron microscope FEIQVANT600, the acceleration voltage is 15 kV, the working range is from 15 mm to 17.2 mm, and the scale bar is from 10 μm to 200 μm. Phase analysis was performed on the D/MAX-2500 X-ray diffractometer. The composition of the reaction interface was analysed by the Link ISIS 6498 energy spectrum analysis system. The macroscopic surface of the interfacial reaction is shown in Figure 1.

## 3. Results

### 3.1. Experimental Results

**Figure 2:**
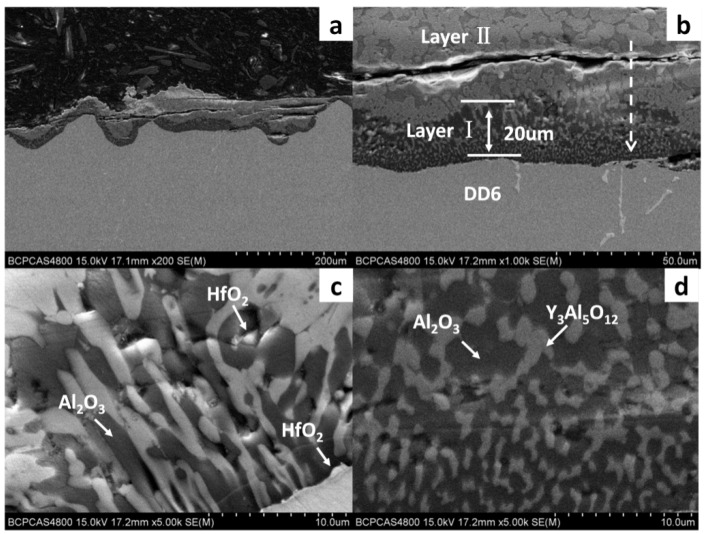
The interfacial reaction between DD6 and Y_2_O_3_ mould: (**a**) cross-Section of alloy; (**b**–**d**) high magnification.

**Figure 3:**
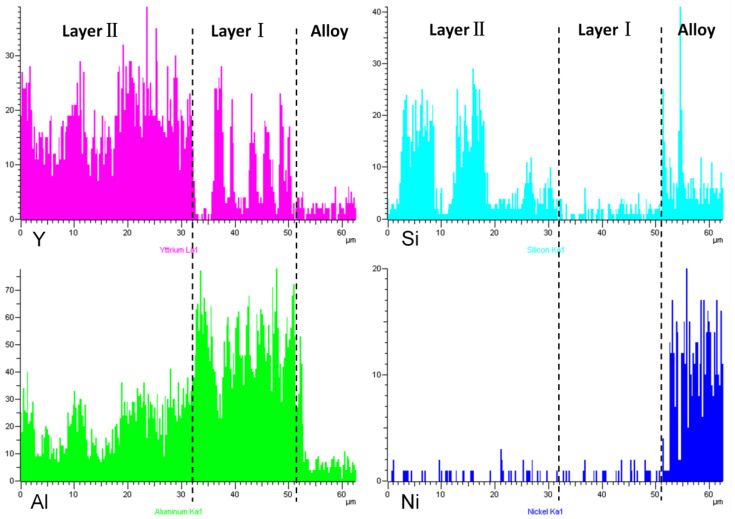
The EDS line scan of interfacial reaction layer between DD6 alloy and Y_2_O_3_ mould.

**Figure 4:**
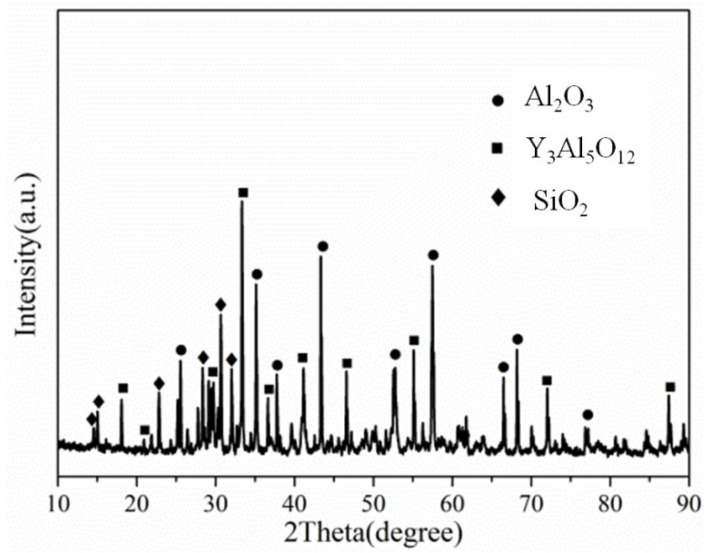
The XRD of interfacial reaction layer II.

Figure 2 shows a cross-sectional view of the reaction interface between the DD6 alloy and the yttrium oxide prime layer of the mould at a pouring temperature of 1530 °C. As shown in Figure 2a, the sand burning occurs on the surface of the DD6 alloy heavily. Although there is no apparent HfO_2_ reaction product layer, a small amount of HfO_2_ is found in some areas of the alloy boundary (shown in Figure 2c), indicating that a chemical reaction has occurred: Hf + SiO_2_ → HfO_2_ + Si, where Hf is from the DD6 alloy and SiO_2_ is mainly from the binder colloidal silica. According to the different morphology of the reaction layer, the interface between DD6 alloy and the yttrium oxide prime layer of the mould can be divided into two reaction layers: I and II. The EDS line scan through the reaction layer is shown in Figure 2b, and the results are shown in Figure 3. The Si content in the reaction layer I decreased significantly lower than in the layer II, while the Si content in the alloy increased (shown in Figure 3); at the same time, the Al content in the reaction layer of the entire mould increased, reaching the highest in the layer I, and then decreased with the increase of the interfacial distance, but consistently higher than the Al content in the alloy (shown in Figure 3). This indicates that a chemical reaction occurred: Al + SiO_2_ → Al_2_O_3_ + Si, where Al comes from the DD6 alloy and SiO_2_ comes from the binder colloidal silica. During the reaction, a large amount of SiO_2_ in the layer I was consumed which significantly reduced the Si content, while the Al_2_O_3_ generated by the reaction remained in the mould, so that the Al content increased. At a high temperature of about 1500 °C, the reaction of Al_2_O_3_ and the mould prime layer Y_2_O_3_, the solid phase reaction to form Y/Al solid solution, EDS analysis showed that the Y/Al ratio of the white particles in Figure 2d is about 0.6, so it is preliminarily determined that the Y_2_O_3_–Al_2_O_3_ reaction generates Y_3_Al_5_O_12_. Therefore, the layer I of the interfacial reaction product is mainly composed of Al_2_O_3_ and Y_3_Al_5_O_12_ particles, and the SiO_2_ content is shallow, while the interfacial reaction layer II is mainly composed of SiO_2_, Al_2_O_3_ and Y_3_Al_5_O_12_ particles as is showed in Figure 4.

**Figure 5:**
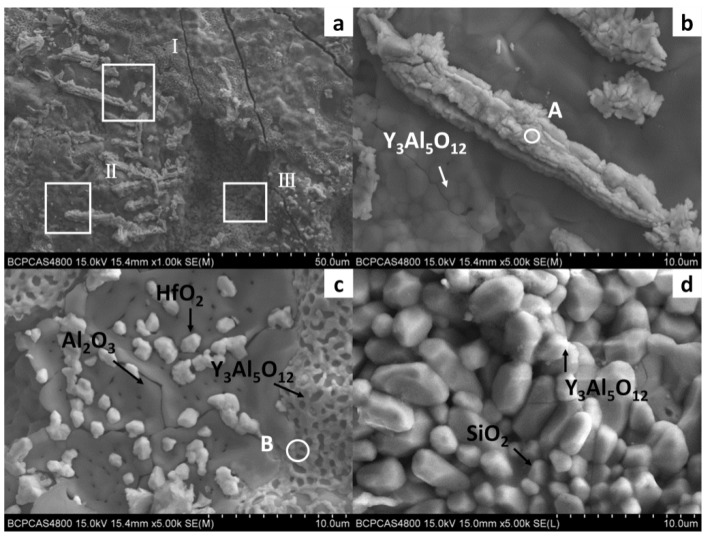
The interfacial reaction between DD6 and Y_2_O_3_ mould: (**a**) cross-Section of mould; (**b**) high magnification of zone I; (**c**) high magnification of zone II; (**d**) high magnification of zone III.

**Figure 6:**
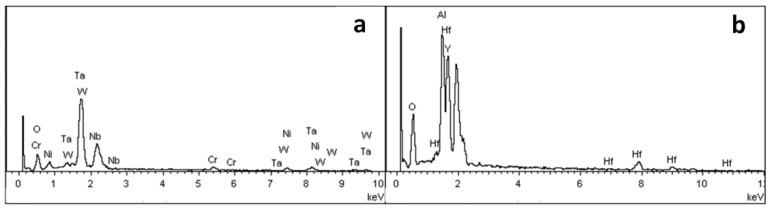
The EDS spectra: (**a**) point A in Figure 5b, and (**b**) point B in Figure 5c.

In order to further study the morphology and distribution of oxide particles in the reaction layer, the inner surface of the cast mould and the surface of the DD6 casting after pouring were micro-observed, as shown in Figure 5. The morphology of the inner surface of the yttrium oxide prime layer of the mould after casting at 1530 °C is shown in Figure 5. The inner surface of the mould forms an irregular two-dimensional network of Al_2_O_3_ (shown in Figure 5c), and the white HfO_2_ particles are randomly embedded in the Al_2_O_3_ network. The surface layer Y_3_Al_5_O_12_ is distributed as an irregular network (shown in Figure 5c) or granular (shown in Figure 5d). The EDS analysis of the Y_3_Al_5_O_12_ network region (shown in Figure 6b) shows that there is also a specific HfO_2_ distribution, but no SiO_2_ is present, indicating that the surfaces shown in Figure 5b,c correspond to the reaction I region of Figure 2. The amorphous SiO_2_ was found in the Y_3_Al_5_O_12_ particle gap, indicating that the surface in Figure 5d corresponds to the reaction region II of Figure 2. In addition, Nb- and Ta-enriched oxides were also found on the inner surface of the yttrium oxide prime layer. As shown in Figure 5b, the Nb- and Ta-enriched oxides were granular in a layer of granular form and rich in the extended structure; the Ta/Nb ratio and the presence of Cr, W, and other elements may be the reason why the morphology of the oxide is different from the interface Nb and Ta oxides of other surface materials. The EDS analysis in Figure 6a confirmed the elements composition.

**Figure 7:**
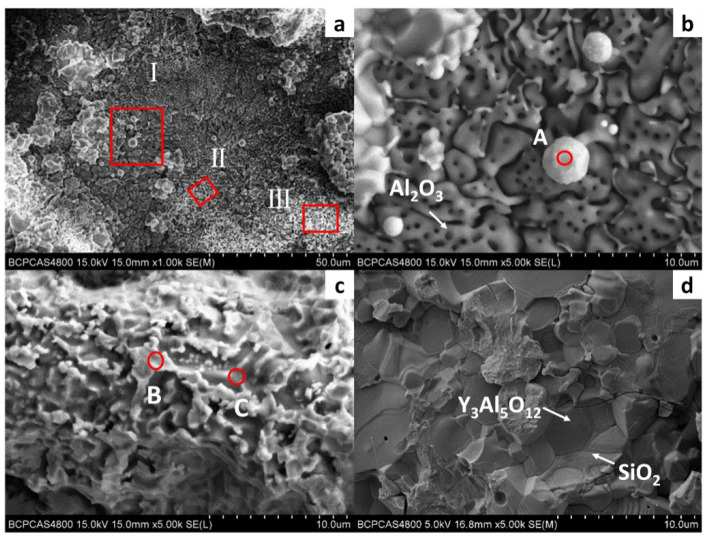
The interfacial reaction between DD6 and Y_2_O_3_mould: (**a**) surface of alloy; (**b**) high magnification of zone I; (**c**) high magnification of zone II; (**d**) high magnification of zone III.

**Figure 8:**
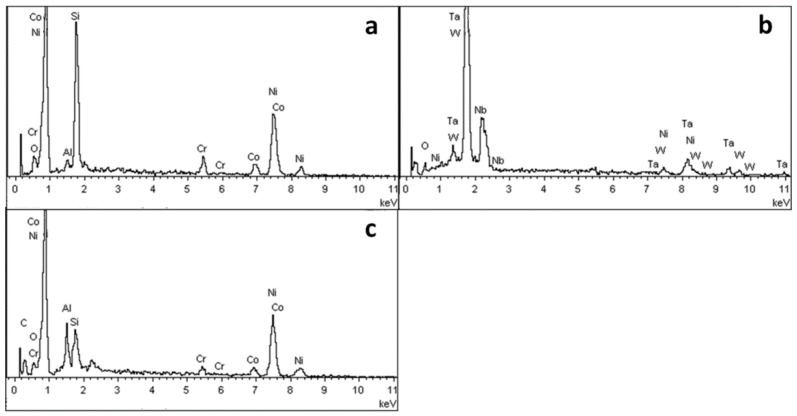
The EDS spectra: (**a**) point A in Figure 7b, (**b**) point B in Figure 7c, (**c**) point C in Figure 7c.

Figure 7 shows the surface morphology of the DD6 casting after pouring at 1530 °C. Combined with EDS analysis, it can be seen that the reaction layer covers the surface of the casting. Similar to the results shown in Figure 5, Al_2_O_3_ in the reaction layer is still an irregular network (shown in Figure 7b), and the Y_3_Al_5_O_12_ particle is surrounded by amorphous SiO_2_ (shown in Figure 7d). Different from the results shown in Figure 5, the O content is reduced in the region similar to Nb and Ta oxides in the reaction layer. That is, the region is more enriched by Nb, Ta, W and other metals, and the Ta/Nb ratio is increased. The Al and Si content in the Ni alloy after the segregation of the nearby components is significantly higher than that of the DD6 alloy, which further shows that the interfacial reaction process of Al oxidation is accompanied by segregation of Nb, Ta, W and other components. However, the relationship between the interfacial reaction and the segregation of Nb, Ta and other components and Nb. The formation mechanism of Ta oxides needs to be further studied. In addition, a small number of white spherical particles were observed in Figure 7b, and the EDS results shown in Figure 8a showed that they were Ni alloys with high Si content after the interfacial reaction, which may be the reason for the sharp increase in Si content near the interface of Figure 5b.

### 3.2. Analysis and Discussion

#### 3.2.1. Mechanism Analysis of Interfacial Reactions

**Figure 9:**
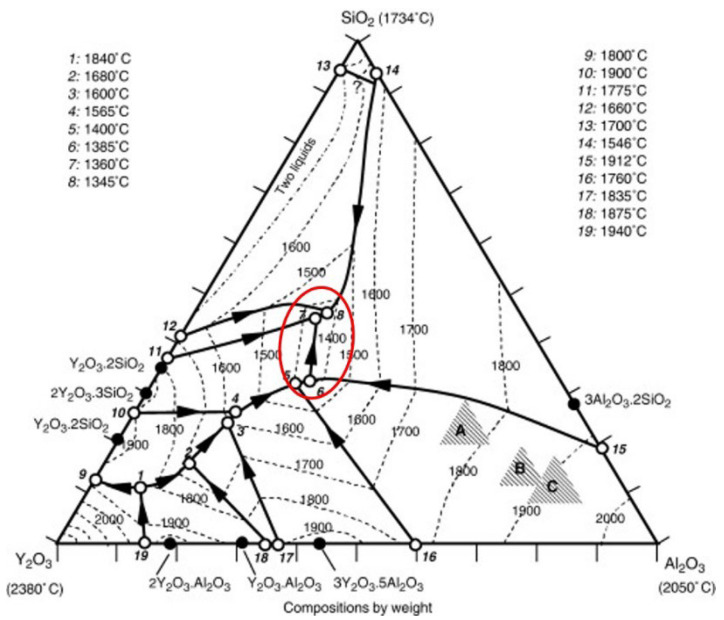
Y_2_O_3_–Al_2_O_3_–SiO_2_ ternary phase diagram.

In addition to Y_2_O_3_, the yttria-type mould prime layer material has about 10% SiO_2_, and this part of SiO_2_ comes from the binder colloidal silica. The alloy contains about 5% of the Al element; therefore, it can react with SiO_2_ in the mould prime layer and other impurity elements to produce Al_2_O_3_. As seen from the ternary phase diagram of Figure 9: Y_2_O_3_–Al_2_O_3_–SiO_2_, there are four low conversion points (points 5, 6, 7, 8) in the 1350–1400 °C range. Since the pouring temperature is 1530 °C, with the interfacial reaction, the proportion of Al_2_O_3_ generated by the reaction and the mould prime layer Y_2_O_3_–SiO_2_ gradually changes. After reaching the appropriate proportion, the liquid phase is gradually formed and diffuses to the alloy side, and even the liquid phase flows, gradually eroding the alloy blades. After pouring, the reaction product that diffuses into the metal side solidifies and peels off with the mould removal process, leaving a reaction pit on the surface of the blade. The occurrence of the yttrium-type mould interfacial reaction is not because the Y_2_O_3_ powder itself has an interfacial reaction with the alloying elements, but due to the formation of Y_2_O_3_–Al_2_O_3_–SiO_2_ ternary low-melts, after complex physicochemical processes, such as element diffusion, displacement reaction, liquid phase flow, etc., finally forms the interfacial reaction surface shown in Figure 1. Avoiding the formation of Y_2_O_3_–Al_2_O_3_–SiO_2_ ternary low-melts can solve the interfacial reaction between DD6 alloy and yttrium oxide mould.

#### 3.2.2. Interfacial Reaction Model

**Figure 10:**
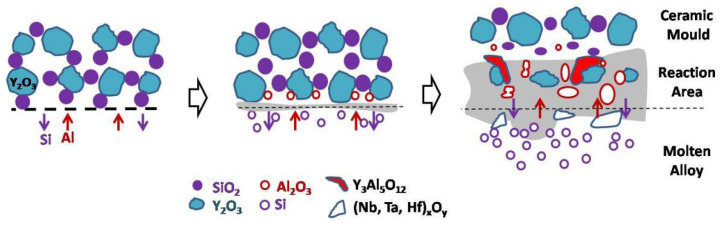
The inter-diffusion mechanism of reaction layer.

The interfacial reaction between the DD6 single crystal superalloy and yttrium oxide mould belongs to the mutual diffusion type. The physical wetting process of the interfacial reaction and the initial formation of the reaction layer is similar to the surface sticky sand type, but due to the different mould materials, the interfacial reaction products are different. The reaction layer growth process is different, resulting in different morphology of the final reaction layer, as shown in Figure 10. The main product of the interfacial reaction layer is still Al_2_O_3_, and the Al_2_O_3_ generated at a high temperature can be cured with the Y_2_O_3_ in the mould to form an yttrium–aluminium solid solution with Y_3_Al_5_O_12_ or other Y/Al ratios. In contrast, the SiO_2_ in the reaction layer is reduced due to the reaction consumption, and the element distribution results of the reaction layer shown in Figure 10 appear. In some areas of the alloy surface, the phenomenon of intensified interfacial reaction may occur due to high-temperature liquid phases such as yttrium–aluminium solid solutions, resulting in the diffusion of the reaction layer to the inside of the alloy to form a pit-like interfacial reaction surface. It is precise due to the mutual diffusion of the reaction layer in the mould and the alloy that the surface of the DD6 alloy specimen poured with yttrium oxide mould is seriously sticky.

## 4. Conclusions

The interfacial reaction between ceramic mould and single crystal superalloy for manufacturing turbine blade were investigated in the present work, and the interfacial reaction mechanism were also discussed in detail. The conclusions are as follows:

- The ceramic mould with the yttrium oxide powder as the prime coating materials had an interfacial reaction with the DD6 alloy, and the main reaction products were HfO_2_, Al_2_O_3_ and Y_3_Al_5_O_12_, while alloy surface suffered undesirable sand fusion;
- The thickness of the reaction layer was over 20 μm. The reaction layer can be divided into two layers, and the layer close to the alloy was mainly composed of Al_2_O_3_ and Y_3_Al_5_O_12_, and the layer was close to the mould composed of SiO_2_, Al_2_O_3_ and Y_3_Al_5_O_12_;
- The interfacial reaction mechanism between Y_2_O_3_ mould and DD6 single crystal superalloy is the formation of Y_2_O_3_–Al_2_O_3_–SiO_2_ ternary low-melts, after complex physicochemical processes, such as element diffusion, displacement reaction, liquid phase flow and so on. Avoiding the formation of Y_2_O_3_–Al_2_O_3_–SiO_2_ ternary low-melts can solve the interfacial reaction between DD6 alloy and yttrium oxide mould.

## Figures and Tables

**Table 1:** Nominal composition of DD6 superalloys (mass fraction%).

C	Cr	Co	W	Mo	Al	Ti
0.001–0.04	3.8–4.8	8.5–9.5	7.0–9.0	1.5–2.5	5.2–6.2	≤0.10
**Fe**	**Nb**	**Ta**	**Re**	**Hf**	**B**	**Ni**
≤0.30	0–1.2	6.0–8.5	1.6–2.4	0.05–0.15	≤0.02	Bal.

**Table 2:** Chemical compositions of the Y_2_O_3_ powders (mass fraction %).

Y_2_O_3_	Al_2_O_3_	Fe_2_O_3_	SiO_2_	MgO
≥99	≤0.03	≤0.03	≤0.01	≤0.01

**Table 3:** Chemical compositions of the white fused alumina powders (mass fraction %).

Al_2_O_3_	SiO_2_	Fe_2_O_3_	Na_2_O
≥99.0	~0.2	~0.1	≤0.3

**Table 4:** Physical and chemical properties of colloidal silica.

SiO_2_%	Na_2_O%	pH	Density/(g/cm^3^)	Kinematic Viscosity/(m^2^/s)
30.12	0.32	10.30	1.20	4.85 × 10^−^^6^

## Data Availability

The data presented in this study are available upon request from the corresponding author.
